# Prevalence and Predictive Factors of Active Myofascial Trigger Points Among Office Workers Using to Visual Display Terminals: A Cross-Sectional Study

**DOI:** 10.1016/j.arrct.2026.100606

**Published:** 2026-02-26

**Authors:** Emilio José Poveda-Pagán, Carlos Lozano-Quijada, Sergio Hernández-Sánchez, José Vicente Toledo-Marhuenda, Eduardo López Pintado, José Vicente Segura-Heras, Jaime Gascón-Jaén

**Affiliations:** a Department of Pathology and Surgery, Miguel Hernández University, Alicante; b Centro de Investigación Traslacional en Fisioterapia, Miguel Hernández University, Alicante; c Clinique de La Source, Lausanne, Suiza; d Centro de Investigación Operativa, Miguel Hernández University, Alicante, Spain

**Keywords:** Low back pain, Neck pain, Prevalence, Rehabilitation, Trigger points

## Abstract

**Objective:**

To determine the prevalence of active myofascial trigger points (MTrPs) in office workers who sit for extended periods and use visual display terminals for ≥4 hours a day. Identify predictive factors associated with MTrP activation.

**Design:**

Cross-sectional study.

**Setting:**

Administration and services departments in municipal settings (January-August 2025).

**Participants:**

A total of 305 municipal volunteer employees (N=305; 133 men: 36.30±8.80y; 172 women: 37.59±9.44y) aged 18-65 years who worked at a video display terminal for ≥4 hours daily were included.

**Interventions:**

Not applicable.

**Main Outcome Measures:**

Participants completed the Neck Disability Index and Oswestry Disability Index. A physiotherapist specialized in myofascial pain assessed bilateral MTrPs in cervical and lumbogluteal muscles using standardized palpation and algometry. Logistic regression models were developed to identify predictive factors, and model performance was evaluated through receiver operating characteristic curve analysis.

**Results:**

Active MTrPs were found in 40% of participants in the right trapezius (25% men; 54% women), 35% in the left trapezius (18% men; 48% women), and 20% in the right sternocleidomastoid (9% men; 28% women), with consistently higher prevalence in women. Neck Disability Index and Oswestry Disability Index scores were identified as significant predictor factors (odds ratio>1), whereas higher pressure pain thresholds were protective (odds ratio<1). The models demonstrated good discriminative ability (area under the curve, 0.74-0.88) and high sensitivity (∼90%).

**Conclusions:**

Active MTrPs show high prevalence among office workers, particularly in women and the cervical region. Disability scores were associated with increased MTrP activation, whereas higher pressure pain thresholds were linked to lower activation. These findings suggest that incorporating algometry into musculoskeletal assessments and strengthening programs for cervical, scapular, and lumbar muscles may help prevent myofascial pain and disability in sedentary workers.

Nonspecific neck and low back pain are among the most prevalent musculoskeletal disorders globally, imposing considerable disability on affected individuals and substantial economic costs through lost workdays, repeated medical consultations, and elevated health care utilization.^1^ Epidemiologic surveys show that in many office-based settings, a large proportion of office workers report experiencing neck pain at some point in their work life. Such pain is now the leading musculoskeletal reason for sickness absence in this population, directly limiting work capacity and productivity.^2^^,^^3^

Office workers are particularly at risk: prolonged exposure to visual display terminals (VDTs) has been significantly associated with heightened neck and shoulder discomfort and the emergence of syndromes commonly referred to as “text-neck” syndromes.^4^, ^5^, ^6^ Additionally, adopting a forward head posture has been recognized as a potential contributing factor to musculoskeletal discomfort. This alignment places additional strain on the cervical spine, which may lead to structural overload, early degenerative changes, or even tissue damage.^7^, ^8^, ^9^ Frequent and prolonged use of electronic devices such as smartphones has also been linked to the onset of neck-related musculoskeletal symptoms, particularly among young adults and university students.^10^^,^^11^

Myofascial trigger point (MTrP) pain is primarily nociceptive but may coexist with nociplastic or neuropathic pain, characterized by altered central nervous system processing, widespread pain, and sleep disturbance.^12^ Regulatory systems are in place at the neuronal junction (both presynaptic and postsynaptic sites), which prevent a dangerous drop in adenosine triphosphate levels or an increase in intracellular calcium levels.^13^ Moreover, central sensitization from nociplastic comorbidities can lower pain thresholds, turning MTrPs into active pain generators.^14^ MTrPs are hyperirritable nodules located within taut bands of skeletal muscle fibers. They may elicit localized or referred pain and can provoke motor, sensory, or autonomic disturbances leading to musculoskeletal dysfunction.^15^^,^^16^ MTrPs are recognized as contributing to cases of cervical and lumbar pain.^17^ In the cervical region, activation of the sternocleidomastoid, levator scapulae, upper trapezius, and infraspinatus can produce a distinctive pattern of referred pain to the neck and shoulder. Conversely, lumbar pain syndromes frequently involve MTrPs in the quadratus lumborum, gluteal musculature, and piriformis, generating referred pain into the lower back and buttock.^18^

Although manual palpation remains the reference standard for identifying MTrPs and validating emerging diagnostic technologies, systematic reviews have demonstrated variable interexaminer reliability, casting doubt on the consistency of palpation as a stand-alone assessment.^19^^,^^20^ This variability underscores the need to refine clinical criteria and establish evidence-based protocols for MTrP detection. Moreover, pressure pain threshold (PPT), assessed via algometry, is a critical quantitative sensory test used to evaluate mechanical hypersensitivity, serving as an objective biomarker for local pain and the presence of central sensitization.^21^

This cross-sectional observational study aimed to determine the prevalence of active MTrPs in employees who spend ≥4 hours per day seated at VDTs and to characterize the clinical features of MTrPs, including taut band, spot tenderness, referred pain, familiar pain, local twitch response, and to quantify mechanical hypersensitivity through PPT assessment.^22^ By identifying which combinations of palpation-based signs most accurately correspond with myofascial pain presentations, the study sought to inform the development of a standardized, evidence-based framework for MTrP assessment within musculoskeletal physiotherapy practice. We hypothesized that a high proportion of office employees would present at least one active MTrP in the upper trapezius or levator scapulae muscles.

## Methods

### Study design

A cross-sectional observational study was conducted in accordance with the Strengthening the Reporting of Observational Studies in Epidemiology Statement: guidelines for reporting observational studies.^23^ Ethical approval was granted by the Ethics Committee of the Miguel Hernández University of Elche (DPC.CLQ.241104). All procedures conformed to the Declaration of Helsinki, and written informed consent was obtained from every participant before data collection.

### Participants

A total of 305 municipal employees (133 men and 172 women) voluntarily participated after an internal email announcement circulated through the Administration and Services departments of Sant Joan d’Alacant Town Council, Spain. Data collection was conducted between January and August 2025. To be included, workers had to be aged 18-65 years and spend ≥4 hours per day at a visual display terminal.

Participants were excluded if they presented with any neurologic disease, acute cervical or lumbar trauma in the previous month, current infection, malignancy, or pregnancy. Those who had taken analgesics within the past 24 hours, had a diagnosed psychological disorder (assessed via self-reported medical history) or substance abuse, or had undergone cervical or lumbar spine surgery were also excluded. In addition, an independent musculoskeletal physician screened all volunteers, and individuals showing clinically relevant limitations in cervical or lumbar range of motion were also excluded.

The physiotherapist performing the physical examination with >20 years of clinical experience was blinded to the participants’ clinical history and questionnaire scores (Neck Disability Index [NDI]/Oswestry Disability Index [ODI]) to prevent detection bias. An additional physiotherapist supported the study by documenting the measurements and guiding participants through each examination step.

### Experimental protocol

All evaluations were performed in a temperature-controlled laboratory at the Sant Joan d’Alacant campus of Miguel Hernández University. Sample randomization was performed using Microsoft Excel (Microsoft Corp.) to minimize systematic bias.

### Procedure for the assessment of MTrPs

The data collection was conducted at the Miguel Hernández University laboratories in Sant Joan d’Alacant. First, participants were fully briefed on the examination procedures for the various MTrPs and written informed consent was obtained. Data were collected using a bespoke questionnaire capturing sociodemographic and anthropometric information (age, sex, height, weight, and dominant side—right or left). Each participant completed the NDI^24^ and the ODI.^25^

**Fig 1:**
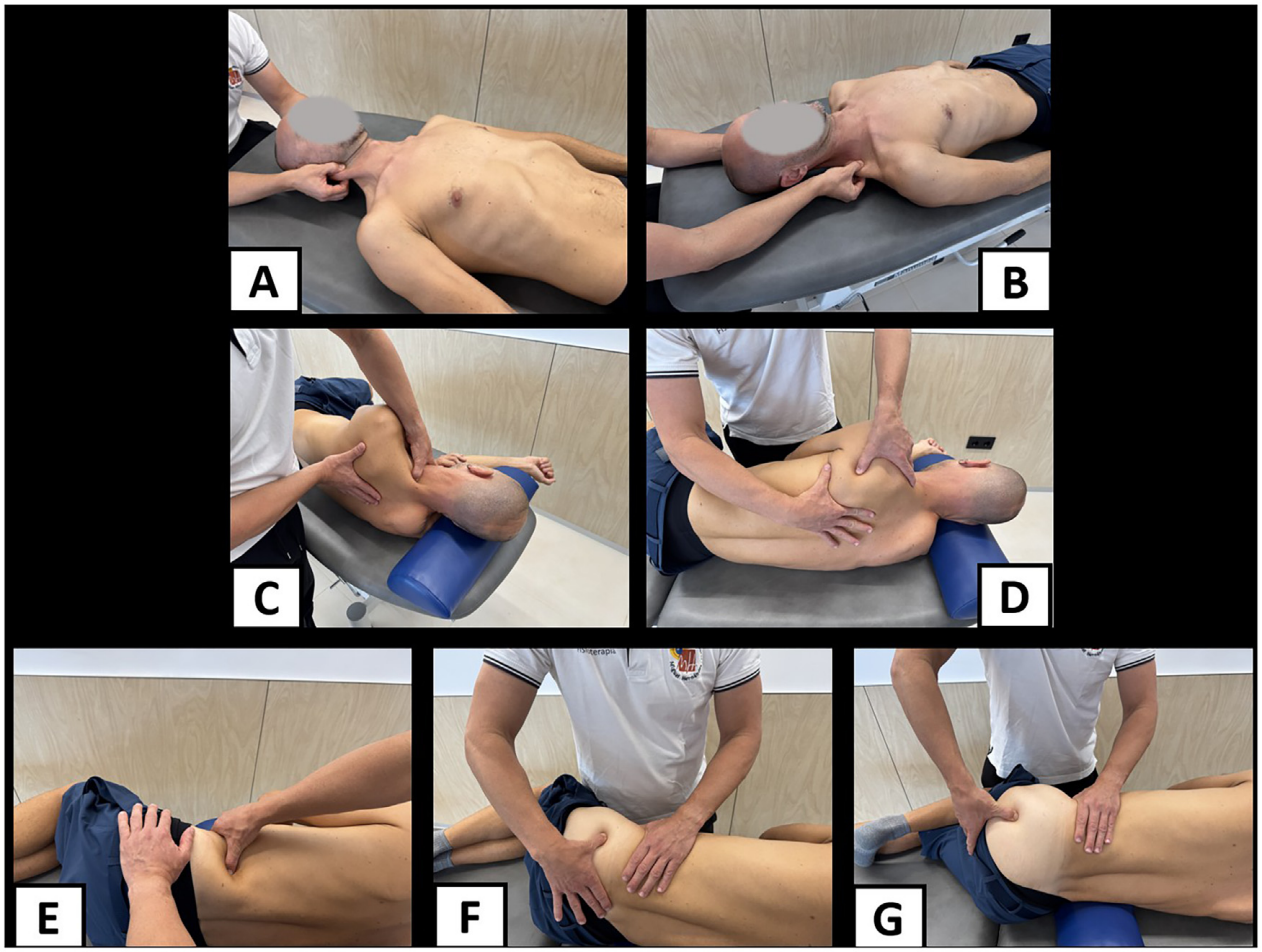
Trigger points examinations in different positions. (A) Sternocleidomastoid (mastoid insertion); (B) upper trapezius (MTrP2); (C) levator scapulae (insertion point); (D) infraspinatus (MTrP2); (E) quadratus lumborum (MTrP1); (F) gluteus medius (MTrP2); and (G) piriformis (MTrP1).

A physiotherapist specializing in MTrP examination assessed each office worker to identify the characteristics of each MTrP. A total of 305 participants were evaluated, with each session lasting 35-40 minutes. Measurements were taken bilaterally (Fig 1) following the procedure described by Donnelly et al^18^: the sternocleidomastoid (mastoid insertion) (Fig 1A) and upper trapezius (MTrP2) (Fig 1B) were first examined on the right side and then on the left. Next, with the participant in the right lateral decubitus position and subsequently in the left lateral decubitus position, the levator scapulae (insertion point) (Fig 1C), infraspinatus (MTrP2) (Fig 1D), quadratus lumborum (MTrP1) (Fig 1E), gluteus medius (MTrP2) (Fig 1F), and piriformis (MTrP1) (Fig 1G) were assessed.

**Fig 2:**
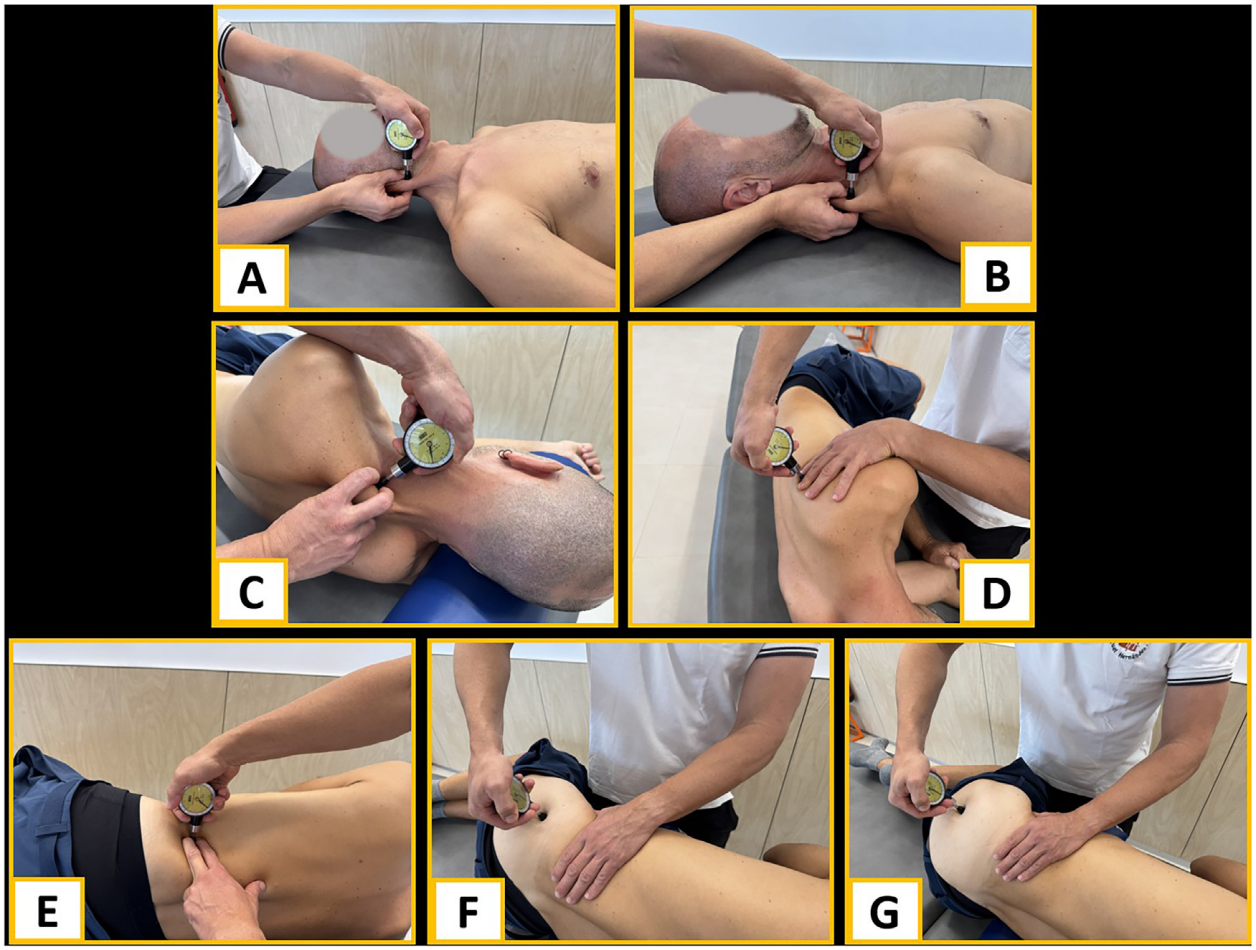
Algometry measurements of the MTrPs. (A) sternocleidomastoid (mastoid insertion); (B) upper trapezius (MTrP2); (C) levator scapulae (insertion point); (D) infraspinatus (MTrP2); (E) quadratus lumborum (MTrP1); (F) gluteus medius (MTrP2); and (G) piriformis (MTrP1).

The parameters were evaluated in the following sequence, applying the updated diagnostic classification proposed by Fernández-de-Las-Peñas et al.^26^ For the purpose of this study, a MTrP was classified as active when 3 mandatory criteria were simultaneously present: a palpable taut band, a hypersensitive spot within that band, and referred pain. Algometry measurements were taken with an analog pressure algometer (Wagner Instruments), which records from 1 kg/cm² up to 10 kg/cm². For each MTrP, 3 readings were obtained, increasing pressure at a rate of 1 kg/cm² per second until the participant first reported pain, at which point that pressure value was noted (fig 2). Between each measurement, a 30-45-second rest interval was provided. The highest of the 3 values was discarded, and the remaining 2 were averaged. Referred pain was then assessed by applying pressure at the participant’s local pain threshold for 5-10 seconds, taking care not to exceed their maximum tolerance.^27^ Familiar pain was confirmed by pressing up to the limit of local pain and asking participants whether this reproduced their usual pain pattern. The local twitch response was characterized by brief, involuntary muscle fiber contractions when rapid, deep transverse friction is applied to an identified MTrP within a taut band.

### Statistical analysis

Continuous variables are expressed as means and standard deviations. Categorical variables are expressed as frequencies and percentages. The chi-square test was used to compare categorical variables and Student’s *t* test to compare continuous variables between groups. Values of *P*<.05 were considered statistically significant. Multiple logistic regression analysis was performed to estimate the probability of presence of active MTrPs in right and left cervical muscles.

To construct the predictive model, the sample was randomly divided into 2 groups: a derivation cohort (2/3 of the sample) used for model development, and a validation cohort (the remaining 1/3) used to test the model’s validity. To construct the model, variables with a *P*<.10 in the univariate model were selected. The odds ratio (OR) and 95% CI were estimated from the regression coefficients. The model was validated using the Hosmer-Lemeshow goodness-of-fit test. Nagelkerke’s R^2^ was used to estimate the proportion of variation explained by the model. Model performance was evaluated using the receiver operating characteristic (ROC) curve and the calculation of the area under the ROC curve. All analyses were performed using the free R software v 4.3.3 (R Core Team).^a^

## Results

Three hundred and five participants were evaluated. The men (n=133) had a mean age of 36.30±8.80 years, and the women (n=172) had a mean age of 37.59±9.44 years. No significant difference was found between the groups (*P*=.223). The mean weight for men (n=133) was 79.90±10.64 kg, which was significantly higher than the mean weight of women (n=172) at 60.40±8.74 kg (*P*<.001). Similarly, men had a significantly greater mean height (176.99±6.78 cm) compared with women (164.09±6.55 cm) (*P*<.001). Regarding disability scores, the mean NDI for men was 5.02±10.19, significantly lower than the 11.97±13.99 mean for women (*P*<.001). Conversely, there was no statistically significant difference in ODI scores between the men (4.09±9.76) and women (5.53±7.79) groups (*P*=.152).

The prevalence of active MTrPs was consistently higher in women than in men (see table 1). The upper trapezius showed the highest rates (40% on the right, 35% on the left), followed by the infraspinatus (23% right, 20% left) and the sternocleidomastoid (20% right, 18% left).

**Table 1:** The prevalence of active MTrPs

Active MTrP	Men (n=133), n (%)	Women (n=172), n (%)	Total (n=305), n (%)
MTrP sternocleidomastoid right	12 (9)	49 (28)	61 (20)
MTrP trapezius right	30 (23)	93 (54)	123 (40)
MTrP levator scapulae right	16 (12)	47 (27)	63 (21)
MTrP infraspinatus right	16 (12)	53 (31)	69 (23)
MTrP quadratus lumborum right	9 (7)	19 (11)	28 (9)
MTrP gluteus medius right	10 (8)	42 (24)	52 (17)
MTrP piriform right	9 (7)	32 (19)	41 (13)
MTrP sternocleidomastoid left	10 (8)	44 (26)	54 (18)
MTrP trapezius left	24 (18)	83 (48)	107 (35)
MTrP levator scapulae left	16 (12)	41 (24)	57 (19)
MTrP infraspinatus left	13 (10)	48 (28)	61 (20)
MTrP quadratus lumborum left	4 (3)	16 (9)	20 (7)
MTrP gluteus medius left	11 (8)	32 (19)	43 (14)
MTrP piriform left	8 (6)	24 (14)	32 (10)

Table 2 shows that in all fitted univariate models, both algometry (PPT) and disability scores (NDI/ODI) were significant predictors. Conversely, age and sex were not individually significant in many cases. For the construction of the multivariate model, we considered factors with an associated *P* value <.10. However, nonsignificant variables were removed from the final model if they did not show a significant effect in the presence of other variables, in order to maintain model parsimony.

**Table 2:** Univariate logistic regression models for predicting the presence of active trigger points in the right and left lumbar muscles (quadratus lumborum, piriformis, and gluteus medius) considering single predictors

MTrP	Algometry	NDI	Age	Sex
Sternocleidomastoid right	<.001	<.001	.762	.001
Trapezius right	<.001	<.001	.803	<.001
Levator scapulae right	<.001	<.001	.039	.264
Infraspinatus right	.001	<.001	.637	.005
Sternocleidomastoid left	<.001	<.001	.733	.003
Trapezius left	<.001	<.001	.049	<.001
Levator scapulae left	<.001	<.001	.231	.201
Infraspinatus left	<.001	<.001	.571	.016
		**ODI**		
Quadratus lumborum right	.001	<.001	.384	.262
Gluteus medius right	<.001	<.001	.336	.001
Pirform right	.001	.019	.152	.043
Quadratus lumborum left	.004	<.001	.392	.064
Gluteus medius left	.001	<.001	<.001	.158
Pirform left	.002	<.001	.105	.110

The results of the multivariate logistic regression model for predicting the presence of active MTrPs in right and left cervical muscles (sternocleidomastoid, trapezius, levator scapulae, and infraspinatus) are shown in table 3, and the results of lumbar muscles (quadratus lumborum, piriformis, and gluteus medius) are shown in table 4. Age was a significant predictor only for the right sternocleidomastoid, with an OR of 1.05 (95% CI, 1.01-1.10), which indicates that each additional year of age increased the chances of having an active MTrP by 5%. On the other hand, NDI and ODI were significant predictors across all muscles, with ORs ranging from 1.04 to 1.10 for NDI and from 1.06 to 1.09 for ODI. With regard to PPT, the ORs were <1 across all muscles (cervical range, 0.08-0.67; lumbar range, 0.19-0.60). Specifically, for the right sternocleidomastoid, the OR was 0.08 (0.01-0.48), indicating that for each unit increase in PPT, the chances of having an active MTrP decreased by ∼92%.

**Table 3:** Multivariate logistic regression model for predicting the presence of active MTrPs in right and left cervical muscles (sternocleidomastoid, trapezius, levator scapulae, and infraspinatus)

		MTrP Sternocleidomastoid Right	MTrP Trapezius Right	MTrP Levator Scapulae Right	MTrP Infraspinatus Right	MTrP Sternocleidomastoid Left	MTrP Trapezius Left	MTrP Levator Scapulae Left	MTrP Infraspinatus Left
Age	OR			1.05 (1.01-1.10)					
NDI	OR	1.10 (1.06-1.15)	1.10 (1.06-1.15)	1.10 (1.05-1.15)	1.04 (1.01-1.07)	1.10 (1.05-1.14)	1.08 (1.04-1.13)	1.10 (1.06-1.15)	1.04 (1.01-1.07)
Algometry	OR	0.08 (0.01-0.48)	0.35 (0.20-0.62)	0.18 (0.08-0.39)	0.67 (0.50-0.90)	0.13 (0.03-0.66)	0.16 (0.07-0.35)	0.21 (0.10-0.43)	0.45 (0.29-0.70)
Sample adjustment (2/3)	R^2^ Nagelkerke	0.44	0.39	0.52	0.16	0.38	0.42	0.48	0.24
	Hosmer-Lemeshow test	0.557	0.274	0.276	0.133	0.203	0.199	0.216	0.369

**Table 4:** Multivariate logistic regression model for predicting the presence of active trigger points in right and left of lumbar muscles (quadratus lumborum, piriform, and gluteus medius)

		MTrP Quadratus Lumborum Right	MTrP Gluteus Medius Right	MTrP Pirform Right	MTrP Quadratus Lumborum Left	MTrP Gluteus Medius Left	MTrP Pirform Left
Age	B	0.016	-0.006	0.043	0.002	0.052	0.032
	OR					1.05 (1.01-1.10)	
Oswestry	OR	1.09 (1.04-1.14)	1.07 (1.03-1.11)	1.06 (1.02-1.10)	1.07 (1.03-1.12)	1.06 (1.02-1.10)	1.09 (1.03-1.15)
Algometry	OR	0.32 (0.17-0.59)	0.49 (0.35-0.70)	0.56 (0.40-0.78)	0.19 (0.06-0.56)	0.58 (0.43-0.79)	0.60 (0.40-0.90)
Sample adjustment (2/3)	R^2^ Nagelkerke	0.33	0.29	0.23	0.36	0.25	0.25
	Hosmer-Lemeshow test	0.963	0.851	0.74	0.4	0.759	0.595

**Fig 3:**
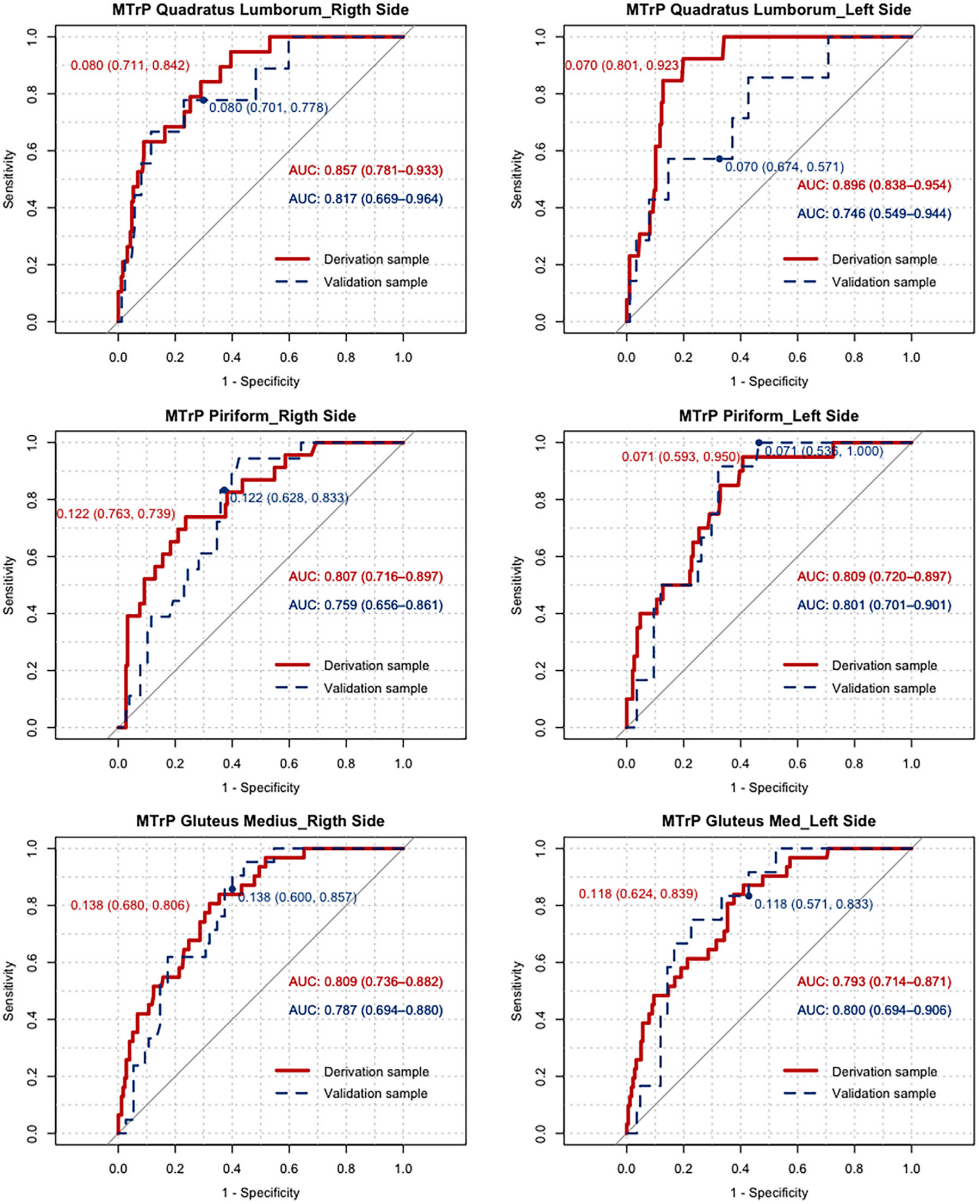
ROC curves for the quadratus lumborum, piriformis, and gluteus medius muscles (bilateral). The legend includes sensitivity, specificity and area under the curve (AUC) with 95% confidence intervals.

**Fig 4:**
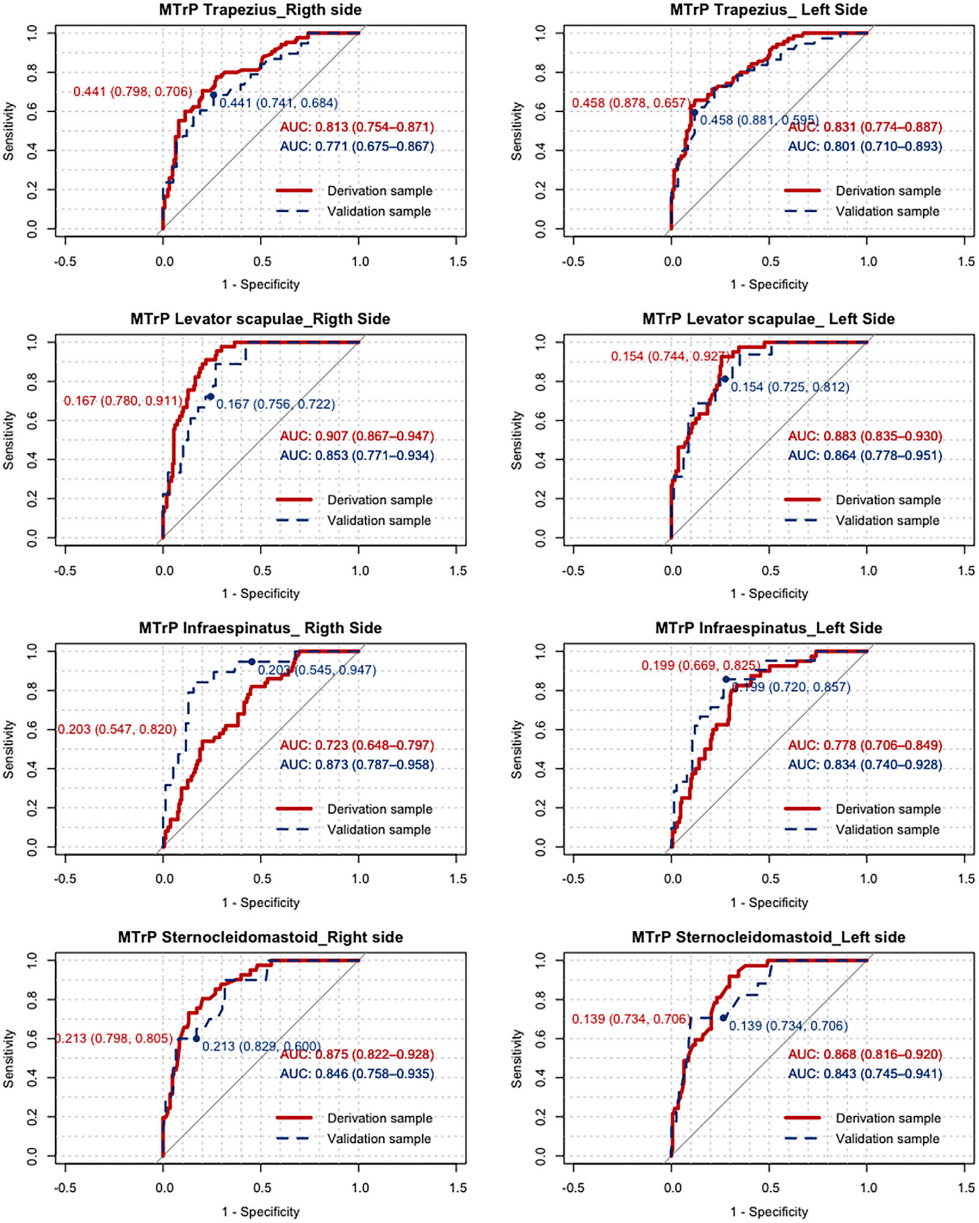
ROC curves for the sternocleidomastoid, trapezius, levator scapulae, and infraspinatus muscles (bilateral). The legend includes sensitivity, specificity and area under the curve (AUC) with 95% confidence intervals.

Finally, the Nagelkerke R² values explained from 16% to 51% of the variance for the cervical muscles (see table 3) and from 23% to 36% of the variance for the lumbar muscles (see table 4). The Hosmer-Lemeshow test *P* values were all >.05, indicating that all models were acceptable. Moreover, the ROC curves showed a high area under the curve, with area under the curve values ranging from 0.74 to 0.82 for the quadratus lumborum, piriformis, and gluteus medius muscles on both sides (fig 3), and from 0.77 to 0.88 for the sternocleidomastoid, trapezius, levator scapulae, and infraspinatus muscles on both sides. Overall, the models demonstrated high sensitivity (∼90%) and moderate-to-high specificity, ranging from 60% to 80% (fig 4).

## Discussion

The main aim of our study was to determine the prevalence of active MTrP in employees who spend ≥4 hours per day seated at VDTs. The findings, after evaluating 305 participants, revealed a high prevalence of active MTrPs among office workers with prolonged VDTs exposure, particularly in women and in the upper trapezius and sternocleidomastoid muscles. These results align with previous research linking repetitive or sustained static postures to increased cervical muscle strain and myofascial pain.^28^

In the cervical region, the highest prevalence of active MTrPs was observed in the upper trapezius and sternocleidomastoid muscles. Clinically, these muscles are among the most commonly affected in patients with nonspecific neck pain, with reported prevalence rates of up to 47% and 30%, respectively.^3^ These findings are also consistent with experimental evidence showing that computer work tasks can induce cervical muscle pain and increased trapezius activity.^2^ In addition, prolonged exposure to VDTs and mobile devices has been identified as a significant risk factor for neck and shoulder pain among office workers.^10^^,^^11^^,^^28^

In the lumbogluteal region, the prevalence of active MTrPs was lower overall, ranging from 7% to 17%, with the piriformis, quadratus lumborum, and gluteus medius being the muscles most frequently affected. These findings are broadly consistent with recent evidence showing that MTrPs are a common feature in patients with low back pain.^29^ Reported prevalence values in the literature vary considerably, with active MTrPs identified in 30%-55% of quadratus lumborum cases, 35%-42% in the piriformis, and up to around 70% within the gluteal region among individuals experiencing low back pain.^29^ These observations highlight the clinical relevance of both cervical and lumbogluteal muscles in nonspecific spinal pain and underscore the importance of incorporating their assessment and management into preventive and therapeutic strategies.

Experimental research supports the physiological basis for the development of MTrPs during prolonged seated computer work. Hoyle et al^30^ demonstrated that even 1 hour of continuous typing under low-level static exertion conditions led to the redevelopment of MTrPs in the upper trapezius, together with increased discomfort and reduced motor unit rotation, indicating local muscle overload. These findings complement epidemiological evidence from Baradaran Mahdavi et al,^31^ who reported that sedentary behavior and prolonged sitting are significant risk factors for low back pain. Together, these results reinforce the notion that sustained static postures and reduced muscular recovery inherent to office work may facilitate the formation and persistence of active MTrPs across cervical and lumbogluteal regions.

A consistently higher prevalence of active MTrPs was observed among women office workers across both cervical and lumbogluteal regions. This sex-related difference is consistent with previous reports suggesting greater susceptibility in women. Beyond hormonal influences and lower pain thresholds, this may be linked to specific biomechanical factors, such as anthropometric mismatches with standard office furniture designed for male averages, which can lead to increased muscle activation. Furthermore, psychosocial hypotheses suggest that the “double burden” of professional and domestic responsibilities may result in higher levels of sustained muscle tension as well as more pronounced activation of the hypothalamic-pituitary-adrenal axis, further modulating pain perception and muscle sensitivity.^32^, ^33^, ^34^ Our results agree with another study reporting a high prevalence of central sensitization, particularly in women, and its strong association with pain located near the trunk.^35^

Age did not appear to be a significant predictor in most models, except for its association with the levator scapulae muscle. Both the NDI and the ODI were identified as significant predictors for MTrP activation, as indicated by ORs >1, suggesting that greater perceived disability increases the likelihood of active MTrPs. In contrast, algometry demonstrated a protective effect, with ORs <1, meaning that higher PPT values are associated with a reduced probability of MTrP activation, particularly in the trapezius and levator scapulae muscles. Therefore, to prevent these issues among office workers, strengthening the neck, shoulder, and scapular musculature appears to be an effective strategy for reducing both neck and low-back-related pain and disability.^36^^,^^37^

The ROC curve analysis demonstrated good discriminative ability of the models showing high sensitivity (approaching 90%), indicating good diagnostic accuracy in detecting MTrP activation when present. However, specificity values were comparatively lower, suggesting a reduced ability to correctly identify the absence of active MTrPs. This pattern reflects a diagnostic model that prioritizes sensitivity, favoring detection of potential cases at the expense of some false positives, which may be appropriate for screening purposes in clinical contexts for the neck and lumbar region. Moreover, the use of algometry may enhance clinical prediction accuracy and is also widely used to assess pain modulation.^38^ The PPT, assessed using algometry, serves as a crucial quantitative sensory testing method for objectively evaluating deep tissue tenderness and is highly relevant in the context of low back and neck pain. The algometer allows for the standardized, noninvasive application of pressure, thus quantifying the participant’s mechanical pain sensitivity.^21^^,^^39^

### Study limitations

We performed a cross-sectional study, the design of which precludes the establishment of causal relationships between disability, PPT, and the presence of active MTrPs. The sample consisted exclusively of office employees using VDTs, which may limit the generalizability of the results to other populations. Although both sexes were represented, the higher proportion of women could have influenced prevalence estimates and model behavior.

The recruitment strategy relied on voluntary participation in response to an email invitation. This may have introduced a volunteer bias, as employees who volunteered to participate may have differed in terms of health awareness or pain perception compared with nonrespondents.

Another potential limitation was the MTrP diagnosis based on manual palpation. Although this is the clinical gold standard, it is characterized by variable interrater reliability, as documented in previous literature.^18^^,^^19^ Although we addressed this by having the same experienced examiner conduct all assessments to ensure internal consistency, the inherent subjectivity of manual palpation must be acknowledged. Furthermore, other potential confounding factors, such as psychosocial stress and specific ergonomic conditions, were not systematically controlled for and should be considered in future research.

Finally, although the predictive models demonstrated good discriminative capacity and high sensitivity, their moderate specificity suggests a possible overestimation of positive cases.

## Conclusions

This study highlights a high prevalence of active MTrPs in sedentary VDT workers, particularly women, with the trapezius and sternocleidomastoid muscles being most affected. Disability scores and lower PPTs were strong predictors of MTrPs activation, suggesting a potential association with central sensitization in myofascial pain. Clinically, these findings may support the integration of algometry and targeted strengthening programs to potentially improve the management of pain and disability in this population. Further research is needed in this field.

## Supplier

a. R; R Core Team.

## Data statements

The data that support the findings of this study are available from the corresponding author upon request.

## Disclosure

The investigators have no financial or nonfinancial disclosures to make in relation to this project.
